# Mixed venous O_2_ saturation and fluid responsiveness after cardiac or major vascular surgery

**DOI:** 10.1186/1749-8090-8-189

**Published:** 2013-09-22

**Authors:** Arjan N Kuiper, Ronald J Trof, AB Johan Groeneveld

**Affiliations:** 1 Departments of Intensive Care, VU University Medical Centre, Amsterdam, The Netherlands; 2 Departments of Intensive Care, Medisch Spectrum Twente, Enschede, The Netherlands; 3 Departments of Intensive Care, Erasmus Medical Center, Gravendijkwal 230, 3015 CE Rotterdam, The Netherlands

**Keywords:** Fluid responsiveness, Mixed venous oxygen saturation, Pulmonary artery catheter, Global end-diastolic volume index, Transpulmonary thermodilution

## Abstract

**Background:**

It is unclear if and how S_v_O_2_ can serve as an indicator of fluid responsiveness in patients after cardiac or major vascular surgery.

**Methods:**

This was a substudy of a randomized single-blinded clinical trial reported earlier on critically ill patients with clinical hypovolemia after cardiac or major vascular surgery. Colloid fluid loading was done for 90 min, guided by changes in pulmonary artery occlusion pressure (PAOP) or central venous pressure (CVP). Fluid responsiveness was defined as ≥15% increase in cardiac index (CI). Hemodynamics, including transpulmonary dilution-derived global end-diastolic volume index (GEDVI) and global ejection fraction (GEF), were measured and blood samples taken.

**Results:**

Whereas baseline S_v_O_2_ (>70% in 68% of patients) did not differ, the S_v_O_2_ increased in patients responding to fluid loading (≥15% in CI in n = 26) versus those not responding (n = 11; P = 0.03). The increase in GEDVI was also greater in responders (P = 0.005). The area under the receiver operating characteristic curve for fluid responsiveness of changes in S_v_O_2_ was 0.73 (P = 0.007), with an optimal cutoff of 2%, and of those in GEDVI 0.82 (P < 0.001), while the areas did not differ. However, the value of S_v_O_2_ increases to reflect CI increases with fluid loading was greatest when GEF was ≤20% (in 53% of patients).

**Conclusions:**

An increase in S_v_O_2_ ≥2%, irrespective of a relatively high baseline value, can thus be used as a monitor of fluid responsiveness in clinically hypovolemic patients after cardiac or major vascular surgery, particularly in those with systolic cardiac dysfunction. Fluid responsiveness concurs with increased tissue O_2_ delivery.

## Background

Hypovolemia is a common problem after surgery and admission in the intensive care unit (ICU), and may reduce cardiac output and O_2_ delivery relative to tissue needs. Circulatory optimization during and after surgery may variably shorten lengths of stay and lower patient morbidity and infusion of fluids may play an important role herein, whereas fluid overloading may be harmful [1-5]. Current indicators of fluid responsiveness, to predict and monitor a preload-dependent increase in cardiac output upon fluid loading and to prevent harmful overloading, include minimally invasive parameters such as dynamic indices obtained by pulse contour methods to more invasive parameters such as transpulmonary dilution-derived cardiac filling volumes [4-8]. The latter have also been used to estimate cardiac function and to guide postoperative fluid therapy [5,9]. In any case, filling pressures may be less helpful, particularly in mechanically ventilated patients, partly because of confounding airway pressures [6]. Although dynamic indices are considered superior in this respect, their value can be confounded, in turn, by ventilatory and cardiac conditions, independently of preload reserve [7].

Since relatively low mixed venous O_2_ saturations (S_v_O_2_) are associated with postoperative complications, (continuous) monitoring has been used to optimize cardiac output and O_2_ delivery relative to tissue needs (O_2_ consumption) after (cardiovascular) surgery. To this end fluids, red cell concentrates and vasoactive drugs have been infused to improve patient outcomes [1-3,9-12]. However, the value of S_v_O_2_ to predict and monitor a cardiac output response to fluids alone, i.e. fluid responsiveness is unclear. Indeed, fluid loading may be the first step to improve tissue oxygenation but may also decrease hemoglobin concentration by hemodilution, decrease arterial SO_2_ by pulmonary fluid overloading or increase supply-dependent O_2_ consumption, so that these factors may limit increases and thereby confound S_v_O_2_ as a well known, curvilinear indicator of cardiac output [3,9-19]. Nevertheless, intermittent or even continuous S_v_O_2_ measurements are often used to assess tissue oxygenation upon fluid loading although the cardiac response per se is not well recognized and the latter could help to define hypovolemia and prevent harmful fluid overloading. Recently, Giraud et al. [20] suggested that in cardiac patients S_v_O_2_ changes are useful monitors of fluid responsiveness, but the role of cardiac function was unclear. This may be important since impaired function and a relatively low cardiac output may diminish fluid responsiveness. Impaired function may also decrease the predictive value of a relatively low baseline S_v_O_2_ but may augment, because of the curvilinear relationship, the value of rises in S_v_O_2_ to reflect increases in cardiac output, if any, upon fluid loading. Conversely, a normal S_v_O_2_ may not exclude fluid responsiveness, if, as in healthy volunteers and septic patients, the heart operates in the steep part of its function curve even when meeting tissue demands, but this is then expected to only minimally increase S_v_O_2_[19,21].

For the current study, we hypothesized that the value of S_v_O_2_ as a predictor and monitor of fluid responsiveness in critically ill, clinically hypovolemic patients after cardiac or major vascular surgery depends on cardiac function. We compared the value with that of transpulmonary dilution-derived global end-diastolic volume, an indicator of preload and, when taking stroke volume into account, of cardiac function [4,5,8,22-24].

## Methods

This is a substudy of a prospective, randomized, single-center clinical trial, investigating the volume expanding effects of various resuscitation fluids [4]. The clinical trial was not registered in an international database. The Ethics Committee of the Vrije Universiteit Medical Center approved the clinical trial and written informed consent was obtained pre-operatively from each patient. During surgery, patients were taken care of by cardioanesthesists applying routine practise according to their clinical standards for hemodynamic management. We analyzed the effect of colloid fluid loading in patients who had undergone cardiac surgery, with or without applying cardiopulmonary bypass (n = 27), or major vascular surgery (n = 10), and who had completed fluid loading and pulmonary artery catheter-derived measurements up to t = 90 min. Pulmonary artery catheters are routinely used in our surgical practise. Colloid fluid loading was given by modified fluid gelatin 4% (Gelofusin^R^), hydroxyethyl starch (200/0.4, Hemohes^R^, B Braun Medical, Melsungen, Germany) 6% or albumin 5% solutions, which have similar oncotic properties and hemodynamic responses [4]. Inclusion criteria, at enrolment and start of the protocol within 3 h after admission into the ICU, were clinical hypovolemia, arbitrarily defined as a systolic blood pressure <110 mmHg and pulmonary artery occlusion pressure (PAOP) <13 mmHg or CVP <12 mmHg, commonly used triggers for fluid infusion at the bedside [4]. Exclusion criteria were age >75 year, preterminal illness with a life expectancy of less than 24 hours, or known anaphylactic reactions to colloids. All peri-operative care was given by attending physicians who were not involved in the study according to institutional and departmental guidelines.

### Study protocol

The protocol was started after arrival of the patients in the ICU. Demographic characteristics were recorded, including the acute physiology and chronic health evaluation (APACHE-II) score. At baseline (t = 0 min), hemodynamic measurements were performed. Heart rate (HR) and mean arterial pressure (MAP), obtained from a continuously recorded electrocardiogram and a radial artery catheter, respectively, were recorded at t = 0 and 90 min. The global end-diastolic volume, central venous pressure (CVP) and PAOP were measured every 30 min, from t = 0 to 90 min. Pressures were measured with patients in the supine position after calibration, zeroing to atmospheric pressure and, for PAOP, after proper wedging, at the midchest level and at end-expiration (Tramscope^R^, Marquette, GE, Milwaukee, Wisconsin). For the measurements of cardiac output and global end-diastolic volume, the transpulmonary thermal-dye indicator dilution technique was used in 30 patients [4,5,8,22-24]. These measurements involve (the average of 2–3) central venous injections of 15 mL of ice-cold indocyanine green in 5% glucose solution and concomitant registration of the dilution curves in the femoral artery, by a 3 F catheter equipped with a thermistor (PV 2024, Pulsion Medical Systems, Munich, Germany). These measurements are relatively insensitive to cardiac valve insufficiencies [22]. The catheter was inserted at the end of surgery via a 4 F introducing sheath (Arrow, Reading, USA) and connected to a bedside computer (COLD Z-021, Pulsion Medical Systems, Munich, Germany. The COLD Z-021 is the precursor to the current pulse contour cardiac output (PiCCO™, Pulsion Medical, Munich, Germany) technique. The global end-diastolic volume represents the volumes of the right and left heart at end-diastole and reflects left ventricular dimensions obtained by echocardiography in the absence of overt right ventricular distension [23]. The global ejection fraction (GEF, normally 25-35%) is defined by the stroke volume/global end-diastolic volume ×4, and can be regarded as a measure of left ventricular systolic function in the absence of right ventricular dysfunction [8]. Reproducibility of transpulmonary measurements is typically within 10%. Arterial and pulmonary artery blood samples were obtained for determinations of hemoglobin/hematocrit (Sysmex SE-9000, Sysmex Corporation, Kobe, Japan) and partial O_2_ pressure/saturation (Rapidlab 865, Bayer Diagnostics, Tarrytown, NY, USA). We calculated O_2_ delivery and O_2_ consumption according to standard formulas. After baseline measurements and blood sampling, fluids were given over 90 min on the basis of the response within predefined limits of increases in PAOP or, in its absence (because of poor wedging in 10 patients), in CVP, according to a previously described protocol yielding safety limits for rapid fluid loading [4]. Up to 200 mL of fluid were given every 10 min, provided that the increase in filling pressures upon fluid loading did not exceed critical values, and this policy has been proven safe in previous studies (i.e. not evoking pulmonary edema) [4]. The maximum amount of fluid infused was 1800 mL. Measurements were done and blood was sampled at t = 90 min. Concomitant vasoactive and sedative drug treatment and ventilatory settings remained unchanged. Indeed, all patients received volume-controlled mechanical ventilation and positive end-expiratory pressure (PEEP). Drainage of blood was <50 mL/hour in all patients, and no patient underwent repeated surgery for bleeding within 12 hours post surgery.

### Calculations and statistical analysis

Parameters were indexed to body surface area (BSA), yielding cardiac index (CI, L/min/m^2^), stroke volume index (SVI, mL/m^2^), O_2_ delivery index (DO_2_I, mL/min/m^2^), O_2_ consumption index (VO_2_I, mL/min/m^2^) and global end-diastolic volume index (GEDVI, mL/m^2^). A GEF <20% was relatively arbitrarily considered to indicate impaired systolic cardiac function [8]. Fluid responsiveness was defined as a ≥15% increase in CI or SVI during fluid administration between t = 0-90 min in agreement with the literature [6-8]. We used the kappa statistic to evaluate concordance of fluid responsiveness by CI and SVI. We used robust non-parametric statistics, because of the relatively small numbers and in spite of mostly normal distributions (Kolmogorov-Smirnov test P > 0.05). The Wilcoxon signed rank test was used for changes in the entire group and the Mann Whitney U test for differences between groups. To investigate correlations between the variables we used partial Pearson correlation coefficients, taking fluid volume and type into account. To evaluate the effect of GEF on fluid response indicators and their first order interaction, we performed generalized estimating equations (GEE). This was also done to relate S_v_O_2_ changes, baseline S_v_O_2_ and their interaction to responsiveness. We used the area under the receiver operating characteristic curves (AUC ROC) (95% confidence interval) to study the value of variables as indicators of fluid responsiveness. AUC ROC curves were compared and sensitivity, specificity, positive and negative predictive values of the optimal cutoff values were calculated (Medcalc^R^, Mariakerke, Belgium). A value of P < 0.05 was considered statistically significant and exact P values are given unless <0.001. Data are summarized as mean ± standard deviation (SD).

## Results

### Study population

Twenty-six (70%) of 37 patients were responders according to CI and 23 (62%) according to SVI increase ≥15% (kappa 0.82). Table 1 shows the comparable baseline characteristics of responder and non-responders.

**Table 1:** Patient characteristics

	**Responder (≥15% in CI increase) n = 26**	**Non-responder**	**P**
		**n = 11**	
Sex, m/f	19/7	10/1	
Age, year	62 (8)	66 (6)	0.14
Weight, kg	81.2 (13.4)	81.1 (9.9)	0.67
Height, m	1.73 (0.09)	1.73 (0.08)	0.68
BSA, m^2^	1.96 (0.19)	1.95 (0.15)	0.90
Surgery, AVR	2	0	
AVR + CABG	3	0	
CABG	12	9	
Closure ASD	1	0	
Off pump	10	6	
Infrarenal aortic aneurysm	4	2	
Thoraco-abdominal aortic aneurysm	4	0	

Mean (SD) or number, where appropriate.

### Cardiac, O_2_-related variables and fluid responsiveness, dependent on systolic cardiac function

Baseline CI and SVI were lower in responders. Fluid loading increased CI/SVI and DO_2_I in responders, and filling pressures, MAP, MPAP and VO_2_I in both responders and non-responders (Tables 2 and 3). Infusion decreased hemoglobin in both responders and non-responders whereas the increase in GEDVI was greater in the former. Fluid loading resulted in a greater increase in S_v_O_2_ in responders than in non-responders, whereas baseline S_v_O_2_ (>70% in 68% of patients) did not differ.

**Table 2:** Hemodynamic variables

	**Responder**	**Non-responder**	**P**
	**n = 26**	**n = 11**	
HR, b/min			
t = 0	74 (11)	67 (7)	0.09
t = 90	75 (11)	70 (11)	0.21
Change	1 (11)	3 (5)	0.73
CI, L/min/m^2^			
t = 0	2.9 (0.6)	3.6 (0.6)	0.005
t = 90^*^	4.0 (0.8)	3.8 (0.6)	0.54
Change	1.1 (0.5)	0.2 (0.2)	na
SVI, mL/m^2^			
t = 0	39 (11)	53 (8)	0.001
t = 90^*^	54 (13)	54 (9)	0.57
Change	14 (12)	1 (4)	<0.001
GEDVI, mL/m^2^			
t = 0	876 (255)	965 (165)	0.13
t = 90^*^	1050 (448)	972 (188)	0.75
Change	173 (264)	8 (103)	0.005
GEF,%			
t = 0	19 (5)	22 (4)	0.13
t = 90^**^	21 (5)	23 (3)	0.40
Change	2 (2)	0 (2)	0.02
PAOP, mm Hg			
t = 0	7 (2)	8 (3)	0.16
t = 90^*^	11 (2)	12 (2)	0.10
Change	4 (2)	4 (2)	0.60
MPAP, mm Hg			
t = 0	15 (4)	18 (5)	0.07
t = 90*	22 (5)	23 (4)	0.27
Change	6 (4)	5 (4)	0.11
CVP, mm Hg			
t = 0	4 (2)	5 (3)	0.32
t = 90^*^	7 (2)	7 (3)	0.95
Change	3 (2)	2 (2)	0.43
MAP, mm Hg			
t = 0	76 (13)	73 (11)	0.27
t = 90^*^	88 (15)	79 (11)	0.10
Change	11 (11)	5 (16)	0.26
Fluid, mL t = 0-90	1575 (232)	1332 (354)	0.05
Type of fluid			
Albumin 5%	10	3	0.77
Gelatin 4%	7	4	
HES200 6%	9	4	

Mean (SD) for responders and non-responders (according to 15% CI increase) or number of patients, where appropriate; ^*^P < 0.001, ^**^P = 0.002 increase t = 0-90 in whole group (R + NR); *na* not applicable.

**Table 3:** Oxygen-related variables

	**Responder**	**Non-responder**	**P**
	**n = 26**	**n = 11**	
Temperature, °C			
t = 0	35.8 (0.6)	35.8 (0.7)	0.90
t = 90	36.1 (0.7)	36.3 (0.9)	0.58
Change	0.3 (0.4)	0.5 (0.6)	0.74
S_a_O_2,_ %			
t = 0	98 (1)	98 (1)	0.44
t = 90	98 (1)	98 (1)	0.81
Change	0 (1)	1 (1)	0.34
S_v_O_2,_ %			
t = 0	72 (7)	75 (7)	0.16
t = 90	74 (7)	74 (6)	0.76
Change	3 (5)	−1 (6)	0.03
Hb, mmol/L			
t = 0	6.0 (1.1)	5.7 (0.6)	0.32
t = 90^*^	5.1 (0.9)	5.1 (0.5)	0.66
Change	−0.9 (0.5)	−0.6 (0.2)	0.10
DO_2_I, mL/min/m^2^			
t = 0	403 (85)	453 (88)	0.18
t = 90^*^	488 (112)	453 (77)	0.31
Change	84 (74)	0 (61)	0.004
VO_2_I, mL/min/m^2^			
t = 0	120 (30)	122 (52)	0.88
t = 90^**^	143 (50)	139 (39)	0.71
Change	22 (50)	16 (67)	0.97
Lactate, mmol/L			
t = 0	1.5 (0.8)	1.0 (0.4)	0.13
t = 90	1.5 (0.8)	1.2 (0.3)	0.68
Change	0 (0.3)	0.1 (0.2)	0.11

Mean (SD) for responders and non-responders (according to 15% CI increase); ^*^P < 0.001, ^**^P = 0.04, for change t = 0-90 in whole group (R + NR).

Table 4 shows that impaired systolic cardiac function (GEF ≤20%) occurred in 53% of patients after surgery and was characterized by relatively low CI and high GEDVI, CVP, MPAP and MAP. The CI response to fluid loading was greater in patients with low than normal GEF. Responders received slightly more fluids (than non-responders) and fluid responsiveness was paralleled by DO_2_I increases irrespective of GEF. S_v_O_2_ increases particularly occurred in responders with low baseline GEF. Baseline MPAP, CVP, GEDVI and CI were lower, and lactate and increases in GEDVI were higher in responders vs non-responders, irrespective of GEF. Lactate decreased in the low GEF group only.

**Table 4:** Hemodynamic and oxygen-related variables according to global ejection fraction (GEF)

	**GEF >20%**		**GEF ≤20%**		**P baseline: G, R, I**
	**R**	**NR**	**R**	**NR**	**change: G, R, I**
	**n = 7**	**n = 7**	**n = 13**	**n = 3**	
HR, b/min					
t = 0	75 (10)	68 (8)	75 (13)	67 (7)	0.92, 0.11, 0.76
t = 90	82 (7)	71 (12)	73 (13)	70 (11)	0.30, 0.29, 0.20
CI, L/min/m^2^					
t = 0	3.3 (0.5)	3.7 (0.7)	2.7 (0.4)	3.3 (0.6)	0.02, 0.01, 0.74
t = 90	4.2 (0.6)	3.8 (0.6)	3.8 (0.7)	3.7 (0.9)	0.05, na, 0.76
GEDVI, mL/m^2^					
t = 0	719 (164)	895 (145)	968 (259)	1128 (51)	<0.001, 0.002, 0.88
t = 90	794 (191)	914 (193)	1199 (492)	1110 (89)	0.54, 0.03, 0.08
PAOP, mm Hg					
t = 0	8 (1)	8 (3)	6 (3)	10 (n = 1)	1.0, 0.07, 0.004
t = 90	12 (2)	11 (2)	11 (2)	14 (n = 1)	0.19, 0.41, 0.30
MPAP, mm Hg					
t = 0	15 (5)	16 (3)	15 (4)	22 (6)	0.06, 0.02, 0.13
t = 90	21 (4)	22 (5)	21 (5)	24 (3)	0.18, 0.61, 0.60
CVP, mm Hg					
t = 0	3 (3)	3 (2)	5 (2)	8 (1)	<0.001, 0.03, 0.03
t = 90	7 (2)	6 (4)	7 (2)	10 (2)	0.18, 0.72, 0.08
MAP, mm Hg					
t = 0	68 (13)	70 (5)	82 (11)	80 (21)	0.03, 0.89, 0.73
t = 90	78 (11)	79 (11)	93 (13)	75 (15)	0.85, 0.11, 0.09
S_v_O_2,_%					
t = 0	73 (7)	75 (6)	71 (7)	71 (9)	0.22, 0.66, 0.67
t = 90	72 (9)	76 (5)	75 (6)	68 (8)	0.92, 0.04, 0.002
DO_2_I, mL/min/m^2^					
t = 0	440 (62)	450 (90)	397 (95)	457 (122)	0.63, 0.35, 0.49
t = 90	480 (129)	436 (62)	498 (112)	476 (120)	0.06, 0.02, 0.53
VO_2_I, mL/min/m^2^					
t = 0	131 (37)	144 (41)	122 (30)	101 (62)	0.15, 0.82, 0.35
t = 90	142 (65)	129 (42)	144 (52)	147 (37)	0.22, 0.89, 0.41
Lactate, mmol/L					
t = 0	1.4 (0.7)	1.0 (0.3)	1.7 (0.9)	1.0 (0.2)	0.31, 0.004, 0.45
t = 90	1.6 (1.0)	1.1 (0.3)	1.5 (0.8)	1.1 (0.2)	0.005, 0.26, 0.18
Fluid, mL t = 0-90					
	1514 (313)	1429 (400)	1577 (205)	1133 (231)	0.28, 0.01, 0.10

Mean (SD); *G* GEF group, *R* response group, *I* interaction, by generalized estimating equations; *na* not applicable.

### Correlations and multivariable analysis

There was a positive correlation between increases in S_v_O_2_ and percentual increases in SVI or CI (r = 0.34, P = 0.04). Similarly, changes in S_v_O_2_ correlated to changes in DO_2_I (r = 0.43, P = 0.01) and VO_2_I (r = −0.38, P = 0.03). GEDVI and CI changes correlated at r = 0.82, P < 0.001. Indeed, fluid responsiveness was associated with changes in S_v_O_2_ (P = 0.04 by GEE) and their interaction with baseline S_v_O_2_ (P = 0.02), rather than with baseline S_v_O_2_ itself (P = 0.16), so that responsiveness was associated with a greater increase in S_v_O_2_ when relatively low at baseline. In the 25 patients with baseline S_v_O_2_ >70%, increases in S_v_O_2_ were associated with fluid responsiveness (in 17 of them, P = 0.01).

### AUCROC curves

Baseline GEDVI was of predictive value at normal GEF and changes in GEDVI and S_v_O_2_ were of particular value at low GEF. Table 5.

**Table 5:** Receiver operating characteristic curves for predicting and monitoring fluid responsiveness

	**AUC**	**P**	**Cutoff**	**Sens.**	**Spec.**	**PPV**	**NPV**
	**(95% confidence interval)**						
For increase in CI ≥15%							
S_v_O_2,_ %	0.65 (0.44 to 0.86)	0.17	77				
Change in S_v_O_2,_ %	0.73 (0.56 to 0.86)	0.007	2	77	73	87	57
GEDVI, mL/m^2^	0.67 (0.47 to 0.88)	0.10	899	68	70	81	54
Change in GEDVI, mL/m^2^	0.82 (0.65 to 1.0)	<0.001	13	95	60	82	86
For increase in SVI ≥15%							
S_v_O_2,_ %	0.60 (0.40 to 0.81)	0.29	77	87	50	74	70
Change in S_v_O_2,_ %	0.68 (0.50 to 0.82)	0.04	2	78	64	78	64
GEDVI, mL/m^2^	0.58 (0.38 to 0.76)	0.48	899	69	62	69	62
Change in GEDVI, mL/m^2^	0.83 (0.65 to 0.94)	<0.001	92	75	92	92	75
For increase in CI ≥15% at high and low GEF							
Change in S_v_O_2,_ %							
GEF >20%	0.57 (0.23 to 0.92)	0.68	3	0	71	0	42
GEF ≤20%	0.94 (0.81 to 1.0)	<0.001	2	92	100	100	95
GEDVI, mL/m^2^							
GEF >20%	0.80 (0.53 to 1.0)	0.02	755	71	86	83	75
GEF ≤20%	0.81 (0.58 to 1.0)	0.008	1028	75	100	100	50
Change in GEDVI, mL/m^2^							
GEF >20%	0.76 (0.47 to 0.94)	0.10	49	86	71	75	83
GEF ≤20%	0.92 (0.73 to 1.0)	<0.001	92	75	100	100	50

**Figure 1:**
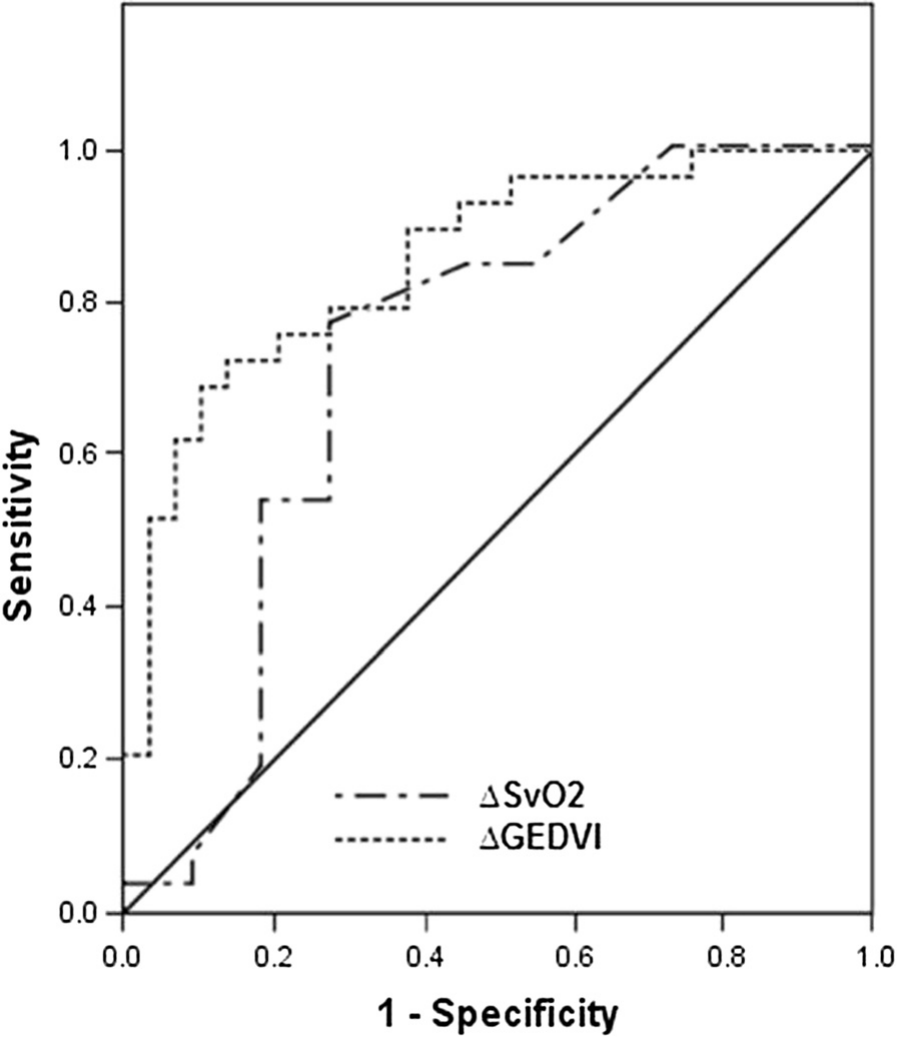
**Receiver operating characteristic curves for fluid responsiveness: changes (∆) in S**_**v**_**O**_**2**_**, AUC 0.73, P = 0.007, and GEDVI, AUC 0.82, P < 0.001**.

The AUROC curves for fluid responsiveness of changes in S_v_O_2_ and GEDVI were significant but did not differ (P = 0.29, Figure 1).

## Discussion

Our study shows that, irrespective of baseline values, an increase of 2% or more in S_v_O_2_ during fluid loading for clinical hypovolemia after cardiac or major vascular surgery indicates fluid responsiveness of the heart, in spite of concomitant hemodilution and a rise in VO_2_I tending to lower S_v_O_2_[11-15,17]. The value is greatest when baseline systolic cardiac function and thus S_v_O_2_ are relatively low, since the percentual increase in CI in the steep part of the cardiac function curve may increase when the latter is displaced downward and to the right.

The patient’s need for fluid loading was judged on clinical grounds and not all patients responded to fluid loading, confirming the superiority of hemodynamic over clinical judgement and the relatively poor predictive value, even at low PEEP, of filling pressures in this respect [6,8]. Our data also suggest that fluid non-responsiveness was not primarily caused by pulmonary hypertension and right ventricular overload, since MPAP never reached values above 35 mm Hg after fluid loading. However, MPAP was slightly higher in patients with low vs normal GEF at baseline, so that increased right ventricular afterload and dysfunction cannot be excluded to have contributed to relatively high GEDVI and non-response to fluids. Also, we cannot exclude a higher left ventricular afterload with higher MAP contributing to a low GEF and cardiac dilatation in some of our patients. The monitoring value of increases in S_v_O_2_ is otherwise similar to that of GEDVI, suggesting that the pulmonary artery catheter and the transpulmonary dilution technique are of similar value in assessing fluid responsiveness, by allowing CI measurements as well as direct indicators of tissue oxygenation and preload reserve, respectively. Insertion of a femoral artery catheter is sometimes considered less invasive than that of a pulmonary artery catheter, however, and the transpulmonary technique also allows monitoring of dynamic indices, which may be of value under some circumstances. In favour of the pulmonary artery catheter on the other hand is the possibility of continuous monitoring of S_v_O_2_ to guide and assess fluid therapy in intervals that arterial O_2_ saturation, haemoglobin and VO_2_I can be assumed to be unchanged [9-15,19]. Moreover, mathematical coupling if variables are taken from the same dilution curves may argue against the seeming superiority (and higher correlation) of GEDVI over S_v_O_2_ increases to reflect CI increases with fluid loading [22]. In contrast, pulmonary catheter-derived CI and S_v_O_2_, measured independently of each other, are not mathematically coupled.

Our finding that an increase in S_v_O_2_ can be used as a monitor of fluid responsiveness in cardiac patients is supported by Giraud et al. [20]. We found that an increase in S_v_O_2_ by 2% or more indicates fluid responsiveness, whereas they [20] found a threshold of 7%. Their baseline S_v_O_2_ was lower than ours, thereby increasing the rise in S_v_O_2_ for a given rise in CI, because of the curvilinear relation between S_v_O_2_ and CI, so that changes in relatively high S_v_O_2_ are less, for given changes in (relatively high) CI, than at low S_v_O_2_ (and thus low CI) [11,12,18]. As in our study, Inomata et al. [14] found a positive correlation between increases in S_v_O_2_ and CI measured with help of a fibreoptic pulmonary artery catheter. However, they studied patients during cardiac surgery and did not specifically look at fluid responses. They observed that when baseline CI was less than 2 L/min/m^2^ the correlation with S_v_O_2_ was better than when it was above 2 L/min/m^2^, probably because the S_v_O_2_ was lower with the former. The effect may be caused, again, by the curvilinear relation between CI and S_v_O_2_ at unchanged arterial O_2_ saturation, hemoglobin and VO_2_I [11,12,18]. This may also explain, together with relatively large fluid responses, the seemingly greater value of an increase in S_v_O_2_ to reflect fluid responsiveness_,_ when baseline cardiac function and thus S_v_O_2_ are relatively low in our study.

In contrast to our observations, Viale et al. [13,15] found no correlation between CI and S_v_O_2_ in the three first postoperative hours after aortic surgery, although they found a positive correlation during surgery. They concluded that lack of correlation was due to a concomitant increase in VO_2_I, that peaked in de second postoperative hour and then returned to preoperative values. Our study started within three hours of arrival at the ICU, and therefore O2 needs may have stabilized, although we cannot exclude that supply-dependent VO2I attenuated the increase in SvO2 with fluid loading in responders, given the inverse correlation between VO2I and SvO2 changes. Otherwise the concomitant increase in DO2I and VO2I can be partly explained by mathematical coupling, in the absence of changes in SvO2. The baseline SvO2 was >70% in many of our patients, and the lactate levels were near normal, particularly in non-responders, and this could together indicate adequacy of tissue oxygenation [1-3,11,12,18,19]. Indeed, the similar baseline VO_2_I among responders and non-responders may indicate similar tissue oxygen needs. The VO_2_I increased with fluid loading in the whole group, but we cannot exclude that this related to increased work performed by the heart following an increased MAP and thus stroke work, even at unchanged CI in non-responders, rather than improved peripheral tissue oxygenation. The lactate levels marginally increased in low GEF patients, possibly consistent with lowered oxygen supply to demand to the heart. In the recent study by Monnet et al. [19] neither baseline nor changes in S_cv_O_2_ were associated with fluid responsiveness, but this may partly relate to S_cv_O_2_ being a poor measure of S_v_O_2_ in their predominantly septic patients, also mostly displaying DO_2_-dependent VO_2_. In agreement with our study, however, the DO_2_ increased in fluid responders [19]. Our results may not be extrapolated to changes in S_cv_O_2_, which may be confounded by changes in mixing of blood from caval veins and coronary sinus [25].

This study carries limitations. We only studied patients after cardiac and vascular surgery and the results may not apply to septic or trauma patients. This study was not designed to improve patient outcomes but to study the circulatory effects of fluid loading, in a posthoc analysis. S_v_O_2_ measurements still require a pulmonary artery catheter and we cannot judge the relative value of S_cv_O_2_ monitoring via central venous catheters, although large differences between the two measurements have been reported after cardiac surgery [10-12,17,20]. The data also suggest that predictive values were independent of the type of fluid loading applied. Finally, the data indicate that responders and non-responders differed in their position on the cardiac function curve, regardless of GEF and position of the curve. The difference is also unlikely caused by an accidental and overall borderline significant difference of 10%, at maximum, in fluid volume load that otherwise exceeded 1 L. The difference may have been evoked by our fluid algorithm limiting fluids when filling pressures increase by more than 5–7 mm Hg [4] per 10 min potentially affecting non-responders more than responders.

## Conclusion

Increases in S_v_O_2_ of 2% or more help to monitor the CI response to fluid loading resulting in increased tissue O_2_ delivery, particularly when systolic cardiac function is impaired in critically ill patients with clinical hypovolemia after cardiac or major vascular surgery. Even though a normal baseline S_v_O_2_ does not predict nor exclude fluid responsiveness, monitoring of S_v_O_2_ may circumvent or supplement thermodilution CI measurements for its assessment.

## Abbreviations

ICU: Intensive care unit; DO2I: O_2_ delivery index; VO2I: O_2_ consumption index; CI: Cardiac index; SVI: Stroke volume index; GEDVI: Global end-diastolic volume index; GEF: Global ejection fraction; PAOP: Pulmonary artery occlusion pressure; CVP: Central venous pressure; MAP: Mean arterial pressure; MPAP: Mean pulmonary artery pressure; HR: Heart rate; SaO2: O_2_ saturation in arterial blood; Hb: Hemoglobin; SvO2: O_2_ saturation in mixed venous blood; ScvO2: O_2_ saturation in central venous blood; PEEP: Positive end-expiratory pressure; BSA: Body surface area; AVR: Aortic valve replacement; CABG: Coronary artery bypass grafting; ASD: Atrial septal defect; HES: Hydroxyethyl starch; AUC: Area under the curve; ROC: Receiver operating characteristic; Sens.: Sensitivity; Spec.: Specificity; PPV: Positive predictive value; NPV: Negative predictive value; R: Responder; NR: Non-responder.

## Competing interests

The authors declare that they have no competing interests.

## Authors’ contributions

ANK, RJT and ABJG equally participated in study design, execution, data analysis and paper preparation. All authors read and approved the final manuscript.
